# Co-targeting the PI3K/mTOR and JAK2 signalling pathways produces synergistic activity against myeloproliferative neoplasms

**DOI:** 10.1111/jcmm.12162

**Published:** 2013-11-17

**Authors:** Niccolò Bartalucci, Lorenzo Tozzi, Costanza Bogani, Serena Martinelli, Giada Rotunno, Jean-Luc Villeval, Alessandro M Vannucchi

**Affiliations:** a Department of Experimental and Clinical Medicine, University of FlorenceFlorence, Italy; b Inserm, U.1009, Institut Gustave Roussy and Université Paris XIVillejuif, France

**Keywords:** JAK2, myeloproliferative disorders, PI3K pathway, *in-vivo*, BEZ235

## Abstract

Aberrant JAK2 signalling plays a central role in myeloproliferative neoplasms (MPN). JAK2 inhibitors have proven to be clinically efficacious, however, they are not mutation-specific and competent enough to suppress neoplastic clonal haematopoiesis. We hypothesized that, by simultaneously targeting multiple activated signalling pathways, MPN could be more effectively treated. To this end we investigated the efficacy of BEZ235, a dual PI3K/mTOR inhibitor, alone and in combination with the JAK1/JAK2 inhibitor ruxolitinib, in different preclinical models of MPN. Single-agent BEZ235 inhibited the proliferation and induced cell cycle arrest and apoptosis of mouse and human *JAK2*V617F mutated cell lines at concentrations significantly lower than those required to inhibit the wild-type counterpart, and preferentially prevented colony formation from JAK2V617F knock-in mice and patients' progenitor cells compared with normal ones. Co-treatment of BEZ235 and ruxolitinib produced significant synergism in all these *in-vitro* models. Co-treatment was also more effective than single drugs in reducing the extent of disease and prolonging survival of immunodeficient mice injected with *JAK2*V617F-mutated Ba/F3-EPOR cells and in reducing spleen size, decreasing reticulocyte count and improving spleen histopathology in conditional JAK2V617F knock-in mice. In conclusion, combined inhibition of PI3K/mTOR and JAK2 signalling may represent a novel therapeutic strategy in MPN.

## Introduction

The chronic myeloproliferative neoplasms (MPN), that include polycythaemia vera (PV), essential thrombocythaemia (ET) and primary myelofibrosis (PMF), originate from a transformed haematopoietic stem or progenitor cell resulting in overproduction of mature blood cells of one or more cell lineages 1. These incurable disorders cause a reduction of life expectancy, particularly in PMF 2, and eventually transform to acute leukaemia. Myeloproliferative neoplasms patients are characterized by a *JAK2*V617F point mutation in 95% of PV and 60% of ET and PMF 3–4; furthermore, 5 or 10% of ET and PMF patients harbour mutations in *MPL*, respectively 5–8. These molecular abnormalities determine a constitutive activation of the JAK/signal transducer and activator of transcription (STAT) signalling pathway as well as of the mitogen activated protein (MAP) kinases/extracellular signal-regulated kinase (ERK) and the phosphoinositide-3-kinase/protein kinase B-AKT (PI3K/AKT) pathways. Aberrant JAK2 activation is largely responsible for the cytokine hypersensitivity and cytokine independent growth of mutant cells, as exemplified by the endogenous erythroid colonies (EEC) typical of PV patients 9,10. Retroviral, transgenic and conditional knock-in (KI) mouse models showed that expression of *JAK2*V617F is sufficient to recapitulate a MPN phenotype 11–14, indicating the central role of constitutive activation of the JAK2/STAT pathway in these pathologies.

Conventional treatment of MPN involves cytotoxic drugs (mainly hydroxyurea), interferon, anagrelide, aspirin and stem cell transplantation in high-risk MF patients 15. The discovery of aberrantly activated JAK2 signalling prompted the development of small molecule inhibitors. In preclinical models, type I ATP-competitive JAK2 inhibitors efficaciously prevented the proliferation of *JAK2*V617F mutant cells *in vitro* and mitigated myeloproliferation in *JAK2*V617F and *MPL*W515L transgenic animals 16,17. Numerous clinical trials with JAK2 inhibitors are being conducted in MPN patients, particularly with myelofibrosis 19,20. These drugs, with some differences in safety profile, almost uniformly showed clinical efficacy with reduction of splenomegaly and improvement of disease-associated symptoms, an effect largely, but not universally 21, ascribed to a reduction in the levels of proinflammatory cytokines 22. Among JAK2 inhibitors, ruxolitinib is the most advanced in development and has been approved by American and European drug regulatory agencies for the treatment of patients with myelofibrosis based on the results of two phase III trials 23,24. However, treatment with ruxolitinib is associated with only modest decrease of V617F allele burden and clinical responses were documented independent of the *JAK2*V617F mutation status. The inability of current JAK2 inhibitors to appreciably reduce the burden of V617F mutated cells may be largely ascribed to the fact that these molecules are not mutation-selective and do not discriminate the mutated from wild-type (wt) protein. Therefore, it is very likely that the doses of JAK2 inhibitors employed, that are constrained by development of dose-dependent thrombocytopaenia and anaemia reflecting the essential role of JAK2 in normal haematopoiesis, are not sufficiently high to abrogate JAK2 signalling to such an extent to induce clonal cell death. Also, it seems unlikely that eradication of the MPN clone can be achieved with the available JAK2 inhibitors, as suggested by the inability of the JAK2 inhibitor TG101348 12 to affect the disease-initiating cell population in *JAK2*V617F KI mice; therefore novel drugs and/or more effective therapeutic strategies need to be sought.

An aberrant activation of the PI3K/Akt pathway has been documented in *JAK2*V617F mutated cells 25,26, V617F transgenic 27 or KI 28 mice and primary MPN cells 29,30. Furthermore, a possible therapeutic relevance of targeting the activated PI3K/Akt pathway is supported by the improvement of splenomegaly and constitutional symptoms observed in MF patients enrolled in a clinical trial with everolimus, a rapamycin-derivative inhibitor (rapalog) of mTOR 31. The aim of this study was to investigate the activity of BEZ235, a dual phosphatidylinositol-3-kinase (PI3K) and mTOR inhibitor with enhanced activity compared with rapalogs, in preclinical models of MPN, alone and in combination with the JAK2 inhibitor ruxolitinib.

## Materials and methods

### Reagents

BEZ235, a dual PI3K/mTOR inhibitor, and ruxolitinib, a JAK1/JAK2 kinase ATP-competitive inhibitor (both from Novartis) were dissolved in 100% DMSO (Sigma-Aldrich, St Louis, MO, USA) to a final stock concentration of 10 mM. Each stock was used only once by diluting in culture medium. Antibodies against phospho(p)-STAT5 (Tyr694), STAT5, p-4EBP1 (Thr70), 4EBP1, p-mTOR (Ser473), mTOR and tubulin were from Cell Signalling Technology (Danvers, MA, USA).

### Cell lines and cell cultures

The HEL, SET2 and K562 human cell lines were purchased from the German Collection of Microorganisms and Cell Cultures (DSMZ, Braunschweig, Germany). Murine Ba/F3 and Ba/F3-EPOR cells expressing *JAK2* wt or *JAK2*V617F (VF) were donated by R. Skoda (Basel, Switzerland) 32. The original cell lines were expanded briefly in culture and aliquots were frozen in 10% DMSO; all experiments were performed within 2 months after thawing. Cell lines were cultured in RPMI 1640 supplemented with 10% foetal bovine serum (FBS; Lonza, Basel, Switzerland; 20% for SET2), 1% penicillin-streptomycin and L-glutamine. WEHI-conditioned medium (10%) was used for propagation of IL-3-dependent Ba/F3 and Ba/F3-EPOR wt cell lines; at the time of experiments with drugs, Ba/F3-EPOR cells were switched to rhEPO (1.0 U/ml).

### Primary human cells

Peripheral blood (PB) or bone marrow (BM) samples were obtained from PV or PMF patients, diagnosed according to the 2008 WHO criteria 33, under a protocol approved by Institutional Review Board of Azienda Ospedaliera-Universitaria Careggi and after obtaining an informed consent. Mononuclear cells were separated using Ficoll Hypaque (Lonza); CD34^+^ cells were immunomagnetically purified (Miltenyi Biotec, Gladbach, Germany). Control CD34^+^ cells were obtained, after an informed consent, from de-identified volunteer donors or were purchased from Lonza.

### Cell proliferation assay

Cells of human and murine cell lines during the logarithmic phase of growth were plated at 2 × 10^4^/well in 96-well plates with increasing concentrations of the drug(s), in triplicate; the proportion of viable cells, normalized to wells containing an equivalent volume of vehicle (DMSO), was determined at 48 hrs using the WST-1 assay (Roche, Indianapolis, IN, USA). This approach was systematically used after preliminary experiments showed comparable results by counting the proportion of trypan blue-positive cells in an automated cell counter (TC10, Bio-Rad, Hercules, CA, USA). The concentration at which a 50% inhibition of cell proliferation occurred (IC_50_) was calculated using the Origin software (V7.5, OriginLab, Northampton, MA, USA).

### Assessment of cell apoptosis and cell cycle analysis

Quantification of apoptotic cells after 48 hrs drug exposure was accomplished by flow cytometry using the Annexin-V-FLUOS Staining kit (Roche, Basel, Switzerland); at least 20,000 events were acquired in a FACS SCAN flow cytometer. For cell cycle distribution analysis, 1 × 10^6^ cells were harvested after an incubation period of 18 hrs, washed twice with PBS, fixed in ethanol 95% containing RNase 10 μg/ml, and stained with propidium iodide 50 μg/ml for 15 min before flow cytometry analysis. Data were processed with Flow-Jo software (Tree Star, Ashland, OR, USA).

### Colony assays for human haematopoietic progenitors

CD34^+^ cells from PV, PMF or control individuals were plated at 1 × 10^3^/ml in methylcellulose (MethoCult; StemCell Technologies, Vancouver, BC, Canada) supplemented with recombinant cytokines (SCF 50 ng/ml, IL-3 10 ng/ml, IL-6 10 ng/ml, GM-CSF 10 ng/ml, G-CSF 10 ng/ml and EPO 1 U/ml) to promote BFU-E and CFU-GM growth. Endogenous erythroid colonies assay was performed by plating 2.5 × 10^5^/ml PB mononuclear cells of PV patients in methylcellulose containing leucocyte-conditioned medium without EPO (cat. no. #04531; StemCell Technologies). For CFU-Mk, 5 × 10^3^/ml CD34^+^ cells were plated in Megacult Collagen medium with lipids (StemCell Technologies) supplemented with Thrombopoietin 50 ng/ml, IL-3 10 ng/ml, IL-6 10 ng/ml. Colonies were counted on day 12–14 using standard criteria.

### Cell lysis and SDS-PAGE western blotting

Harvested cells were resuspended in RIPA lysis buffer (50 mM pH 7.4 Tris-HCl, 150 mM NaCl, 1% NP-40, 1 mM EDTA) containing a proteinase inhibitor cocktail (Halt Protease Inhibitor Cocktail Kit, Pierce, Rockford, IL, USA). An aliquot of cell lysate was used for protein quantification using a BCA kit (Sigma-Aldrich). Thirty-five micrograms of protein lysate were subjected to sodium dodecyl sulphate polyacrylamide gel electrophoresis separation and western blotting onto Immunoblot PVDF membrane (Millipore, Billerica, MA, USA). Membranes were probed with primary antibodies followed by horseradish peroxidase-conjugated anti-Ig secondary antibody (Sigma-Aldrich); immunoreactive proteins were revealed with ECL. Images were acquired using the ImageQuant 350 apparatus (GE Healthcare, Little Chalfont, UK) and densitometric analysis was performed with the ImageQuant 5.2 software. Tubulin was used for normalization.

### Ba/F3 JAK2V617F-luciferin mouse model

All animal procedures were performed according to Italian laws in an animal facility (Di.V.A.L., University of Florence) under humanized conditions. Female SCID beige mice (4–6 weeks; Harlan, Indianapolis, IN, USA) were given 3 × 10^6^ *JAK2*V617F-Ba/F3-EPOR luc^+^ cells (clone 8) 34, kindly provided by T. Radimerski (Novartis, Basel) by tail vein injection. At specified time points thereafter, mice were injected with Xeno Light D-luciferin (Caliper, Waltham, MA, USA) to generate a measurable bioluminescence signal that is proportional to luc^+^ cells; measurement was performed 15 min after luciferin injection using the Photon Imager apparatus (Biospace Lab, Paris, France). Baseline measurement performed on day 6 after luc^+^ cell injection was used to establish individual bioluminescence level, then mice were randomly divided into four treatment cohorts of six mice each having comparable baseline disease burden (vehicle, BEZ235, ruxolitinib, BEZ235 plus ruxolitinib). Drugs were administered daily by gavage. Imaging was performed at weekly intervals after the first drug dose; mice were followed daily for survival and euthanized when they developed hind limb paralysis or became moribund.

### Conditional *JAK2*V617F KI mouse model

Conditional KI mice were obtained using a flex/switch/recombinase strategy, as described 35. Briefly, a mouse embryonic stem (ES) clone containing the homologously recombined V617F targeting vector allele into the *Jak2* wt allele was injected into blastocyst stage embryos to generate L2 chimeric mice (wt phenotype). Chimeras were bred with flippase (FLP recombinase) transgenic mice to remove the FRT flanked selection *Pgk*NeoR cassette and then crossed with transgenic mice expressing the Cre recombinase under the control of *Vav* promoter (*Vav*Cre) 36. The latter match produced a directed excision of the wt exon13 resulting in the transcription of the G1849U mutated mRNA (KI phenotype). These recombined JAK2^V617F/wt^ KI mice were used for all experiments. Three months-aged KI mice received the drugs for indicated periods and were euthanized by CO_2_ inhalation. Blood samples were collected by retro-orbital plexus puncture; blood parameters were measured using the Sysmex XE5000 (Sysmex, Hyogo, Japan) cell counter, while reticulocytes (number per high-power field, HPF) were counted in methylene blue-stained blood smears. The spleen was collected and weighted; to accomplish for variations in body weight at baseline, a spleen index (*i.e*., spleen weight/body weight ×100) was calculated. Cuts of the spleen were fixed in PBS-buffered formalin (4%), paraffin embedded, sectioned and haematoxylin and eosin stained.

For colony assay, bone marrow cells were collected from *JAK2*V617F KI and *JAK2*wt mice and plated at 1.5 × 10^5^/mL in methylcellulose (MethoCult GF M3434, Stem Cell Technologies) supplemented with recombinant cytokines (SCF, mIL-3, hIL-6, hEPO). In experiments testing drug combination a 1:1 ratio of *JAK2*V617F KI and *JAK2*wt bone marrow mice cells (at a 1 × 10^4^/ml) was used. Colonies were enumerated on day 4 using standard criteria.

### Statistical methods

The Mann–Whitney *U* or Fisher test was used for comparison (SPSS software; StatSoft Inc., Tulsa, OK, USA). The level of significance from two-sided tests was *P* < 0.05. The analysis of drug synergism was performed by calculation of the combination index (CI), that is a measure of the interaction between two drugs. The CI was calculated according to the median- effect principle of the Chou and Talalay method using the CalcuSyn software 2.1 (BioSoft, Cambridge, UK) 37. According to this formula, when CI is less than 0.9 the interaction of two drugs is considered synergistic, when CI is 0.9–1.1 the interaction is additive, and when CI is greater than 1.1 the interaction of two drugs results in an antagonist effect.

## Results

### Impairment of cell viability, induction of cell cycle arrest and apoptosis in *JAK2*V617F mutated cell lines exposed to the dual PI3K/mTOR inhibitor BEZ235

**Figure 1:**
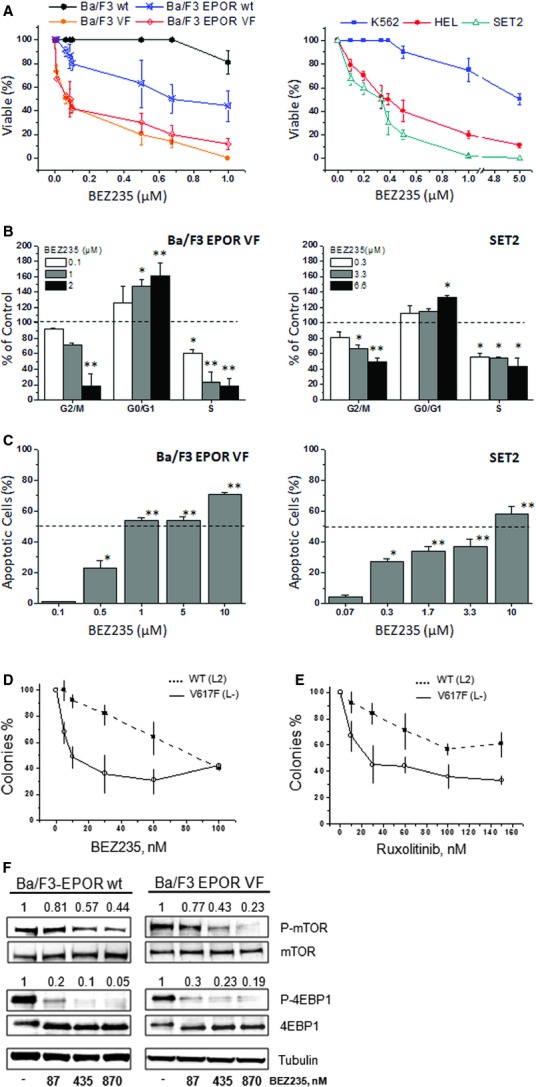
The dual PI3K/mTOR inhibitor BEZ235 reduces the proliferation and induces cell cycle arrest and apoptosis in *JAK2*V617F mutated mouse and human cell lines. (A) *JAK2*V617F (VF) mutated mouse (Ba/F3 and Ba/F3-EPOR) and human (HEL, SET2) cell lines were exposed to varying concentration of BEZ235, and the proportion of viable cells (% of control wells containing vehicle only) was measured at 48 hrs. The wild-type Ba/F3 and Ba/F3-EPOR cells were used as controls in the presence of 10% WEHI-3 conditioned medium and 1.0 U/ml rhEPO, respectively. In case of human cell lines, the BCR/ABL positive K562 cell line was concurrently evaluated for comparison only. Data were expressed as the Means ± SD (*n* = at least five experiments, in triplicate). Details about the IC_50_ and *P* values are reported in the Results section. (B) Effects of BEZ235 on the cell cycle distribution of Ba/F3-EPOR VF and SET2 cells were evaluated after 18 hrs incubation with varying drug amounts. The changes in the proportion of cells in G0/G1, S, and G2/M phase of the cell cycle are indicated as percent of control cells incubated with vehicle only. Data were expressed as the Means ± SD (*n* = at least three experiments). (C) The proportion of Ba/F3-EPOR VF and SET2 cells induced to apoptosis in response to varying amounts of BEZ235 for 48 hrs is shown. Values were expressed as percent of Annexin V-positive cells (Means ± SD of at least three experiments, in duplicate) after subtracting apoptotic cells measured in vehicle-only cultures. (D and E) Progressively increasing amounts of BEZ235 (D) and ruxolitinib (E) were used in clonogenic assay to estimate the relative selectivity of the drugs against haematopoietic progenitors obtained from JAK2V617F KI mice and the JAK2 wild-type counterpart. (F) Expression levels of selected effectors of the PI3K/mTOR pathway in Ba/F3-EPOR VF and the wt counterpart were analysed by western blot in cells that had been exposed for 6 hrs to indicated amounts of BEZ235. The numbers above each lane indicate results of densitometric analysis taking as one the value measured in control cultures incubated with vehicle only. One representative experiment of at least three independent evaluations for the different targets. *P < 0.05; **P < 0.001.

We first evaluated the effects of BEZ235 on the viability of murine and human cell lines expressing the *JAK2*V617F mutation. We found that BEZ235 inhibited the proliferation of Ba/F3 VF and Ba/F3-EPOR VF cells at concentrations significantly lower than the wt counterparts: the IC_50_ value was 87 ± 50 nM in Ba/F3-EPOR VF cells compared with 676 ± 200 nM in the wt counterpart and 64 ± 10 nM in the Ba/F3 VF cells compared with >1000 nM in the wt cells (*P* < 0.01 for both; Fig. 1A). Also the viability of human HEL and SET2 cells was affected by BEZ235 with IC_50_ values of 387 ± 90 nM and 334 ± 40 nM, respectively. Conversely, the BCR/ABL mutated K562 cell line was inhibited at BEZ235 concentrations about 15-fold higher (IC_50_ value = 5000 ± 1000 nM; *P* < 0.01 *versus* SET2 and HEL cells; Fig. 1A). BEZ235 dose-dependently increased the percentage of Ba/F3-EPOR VF cells in G0/G1 phase of the cell cycle, with proportional decrease of the G2/M and S-phase (Fig. 1B); similar effects were observed for SET2 (Fig. 1B) and HEL (data not shown) cell lines. We also found that BEZ235 induced apoptosis in the Ba/F3-EPOR VF and SET2 cell line (Fig. 1C), although higher drug concentrations were required than for proliferation arrest. Finally, to strengthen data supporting a relatively greater selectivity of BEZ235 towards *JAK2*V617F mutated cells, we performed clonogenic assay using bone marrow progenitors from *JAK2*V617F KI mice and the corresponding JAK2 wt genotype. The number of colonies produced in *JAK2*V617F KI cells was inhibited by 50% in presence of 9.4 ± 7 nM BEZ235 compared with 83 ± 1.6 nM for wt cells (Fig. 1D). Colony formation from KI bone marrow cells was also reduced at concentration of ruxolitinib (IC_50_ = 25 ± 1 nM) significantly lower compared with wt cells (IC_50_ ≥ 150 nM; Fig. 1E). We then analysed the expression of selected molecules of the PI3K/mTOR pathway in cells exposed to BEZ235. We found that BEZ235 caused a dose-dependent attenuation of the level of phospho-mTOR that was much more evident in Ba/F3 EPOR VF cells compared with the wt counterpart; on the other hand, the level of phosphorylation of 4EBP1, lying downstream to mTORC1, was similarly reduced in both cells lines (Fig. 1F), consistent with a direct effect of BEZ235 on 4EBP1 38.

### BEZ235 impairs colony formation by haematopoietic progenitor cells of MPN patients and induce apoptosis in CD34^+^ cells

**Figure 2:**
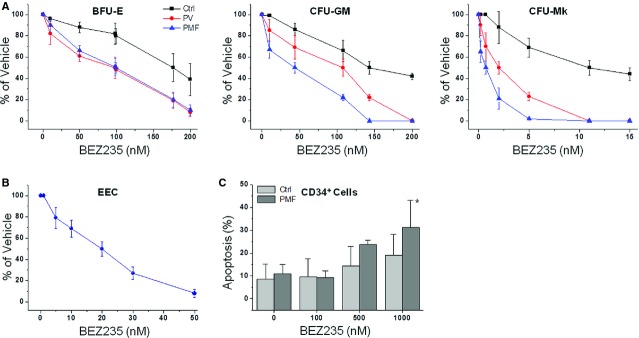
BEZ235 impairs the proliferation of human MPN progenitors, including the endogenous erythroid colonies (EECs), and induces apoptosis of CD34^+^ cells more efficaciously than normal donors' cells. (A) CD34^+^ cells from patients with polycythemia vera (PV) and primary myelofibrosis (PMF) (*n* = 10 each) were plated in duplicate in cytokine-supplemented semisolid media supporting the proliferation of progenitors generating erythroid (BFU-E), myeloid (CFU-GM) and megakaryocytic (CFU-Mk) colonies. Six healthy individuals served as controls (Ctrl). Varying amounts of BEZ235 were added at the beginning of cultures, and colonies were counted by standard criteria after 12–14 days. Values were expressed as the percentage of colonies counted in plates containing vehicle only. Details about the IC_50_ and *P* values are reported in the Results section. (B) Erythropoietin independent erythroid colonies (EEC) were assayed in the absence of EPO starting from peripheral blood mononuclear cells of six PV patients using varying amounts of BEZ235; colonies were counted at day 10 of culture. (C) The effects of varying amounts of BEZ235 on the proportion of apoptotic CD34^+^ cells in culture after 24 hrs; cells were purified from PMF patients and normal individuals (*n* = 3 PMF;*n* = 4, healthy individuals). *P < 0.05.

The efficacy of BEZ235 as a single agent against primary MPN cells was evaluated by clonogenic assay; 10 patients each with PMF (five were *JAK2*V617F mutated) and PV (all *JAK2*V617F mutated), and six healthy donors, were evaluated. Since we found no difference in the response to BEZ235 depending on the *JAK2*V617F mutational status, data were pooled. We found that BEZ235 dose-dependently inhibited colony formation from PMF and PV haematopoietic progenitors at doses significantly lower than normal progenitors (Fig. 2A).The IC_50_ value was 108 ± 7 nM and 44 ± 10 nM for CFU-GM, 98 ± 20 nM and 99 ± 8 nM for BFU-E, 2.1 ± 1.0 nM and 0.7 ± 0.1 nM for CFU-Mk, for PV and PMF patients, respectively, compared with 143 ± 20 nM, 177 ± 50 nM and 11 ± 3 nM for CFU-GM, BFU-E and CFU-Mk from control individuals (all statistically different at *P* < 0.05; Fig. 2A). We also evaluated the effects of BEZ235 on endogenous erythroid colonies in patients with PV (*n* = 6). Endogenous erythroid colonies were dose-dependently inhibited by BEZ235 with an IC_50_ value of 20 ± 10 nM (Fig. 2B). Finally, we determined the effects of BEZ235 on the viability of primary CD34^+^ cells from patients with PMF (*n* = 3). As shown in Figure 2C, BEZ235 at 1 μM induced significantly more apoptosis than in control cells (*P* < 0.05).

### BEZ235 and the JAK2 inhibitor ruxolitinib have synergistic activity against *JAK2*V617F mutated cell lines, haematopoietic progenitors from *JAK2*V617F KI mice and MPN patients

**Figure 3:**
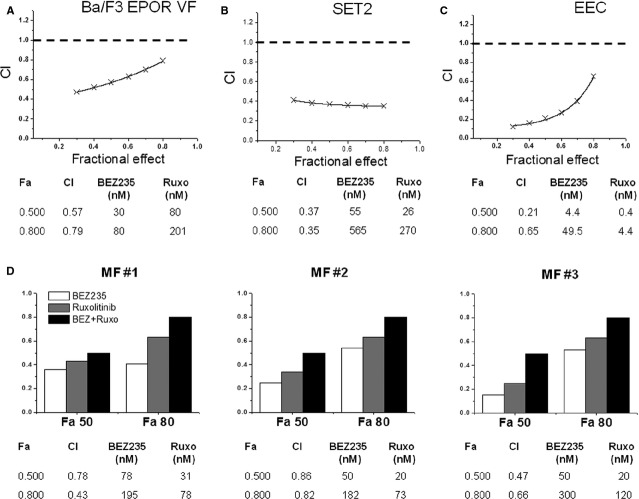
Combination of BEZ235 and ruxolitinib synergistically inhibits *JAK2*V617F mutated Ba/F3-EPOR and SET2 cells, EEC from PV patients, and CD34^+^-derived colonies from PMF patients. (A–C) Progressively increasing amounts of BEZ235 and ruxolitinib were used according to the Chou and Talalay method to estimate the interactions of the drugs in cultures of Ba/F3-EPOR VF cells (A) and SET2 cells (B), and in semisolid media favouring the growth of EEC progenitors from four PV patients (C). End-points were the measure of proliferation inhibition in cultures of Ba/F3-EPOR VF and SET2 cells and the number of EPO-independent erythroid colonies in the EEC assay. A combination Index (CI) was then calculated; a CI < 0.9 indicates that the interaction of the two drugs is synergistic. ‘Fa’ is the cell fraction affected by combined amounts of the two drugs. (D) The effects of combination of BEZ235 and ruxolitinib was also measured in clonogenic assay using CD34^+^ cells obtained from three patients with PMF. Erythroid and myeloid colonies were counted together to calculate the cell fraction affected (only Fa50 and Fa80 values are shown).

**Figure 4:**
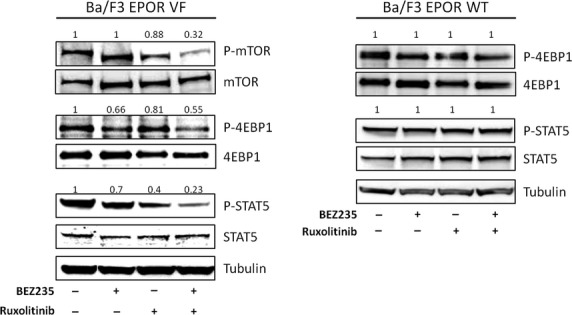
Combination of BEZ235 and ruxolitinib effectively reduces the phosphorylation of selected components of the PI3K/Akt and JAK/STAT signalling pathway. Ba/F3-EPOR VF and wt cells were exposed for 6 hrs to 30 nM BEZ235 and 80 nM ruxolitinib, individually or in combination, and the level of phosphorylated mTOR, 4EBP1 and STAT5 were assayed by western blot. The numbers above each lane indicate results of densitometric analysis taking as one the value measured in cells incubated with vehicle only. One representative of two experiments.

Previous works 18–39 indicated that the JAK2 inhibitor ruxolitinib inhibited the proliferation and induced apoptosis of *JAK2*V617F mutated cells lines. Therefore, we evaluated the effects of co-treatment of ruxolitinib and BEZ235 on the viability of Ba/F3-EPOR VF and SET2 cell lines; to determine potential synergy, we calculated the CI according to Chou and Talalay 37. As shown in Figure 3A, the calculated CI at varying drug combinations indicated strong synergistic activity in the two cell lines. For example, a 50% inhibition of Ba/F3-EPOR VF cell proliferation was obtained at 30 nM BEZ235 and 80 nM ruxolitinib compared with 87 nM and 220 nM, respectively, when the drugs were used alone (Fig. 3A). In case of SET2 cells, 50% inhibition of cell proliferation was obtained using 55 nM BEZ235 and 26 nM ruxolitinib, compared with 334 and 160 nM with each drug alone (Fig. 3B). We next determined the effects of co-treatment of BEZ235 and ruxolitinib in an EEC assay (*n* = 4 PV patients). A 50% inhibition of EEC colony formation was observed at 4.4 nM BEZ235 and 0.4 nM ruxolitinib compared with 20 and 1.8 nM, respectively, for the two drugs alone (Fig. 3C). Efficacy of drug combination was also analysed by measuring the inhibition of GFU-GM and BFU-E colony formation by CD34^+^ cells of three PMF individuals. As shown in Figure 3D, the combination of BEZ235 and ruxolitinib resulted in synergistic inhibition of colony formation compared with single agents. We finally analysed by western blot the level of phosphorylated mTOR, 4EBP1 and STAT5 in Ba/F3-EPOR VF cells exposed to BEZ235 and ruxolitinib alone and in combination (Fig. 4). We found that drug combination produced greater inhibition of phosphorylated mTOR, 4EBP1 and particularly STAT5 compared with single drugs; of note, no significant changes were observed in Ba/F3-EPOR wt cells at the doses employed.

### Combination treatment of BEZ235 with ruxolitinib suppresses JAK2V617F-mediated disease and improves survival in mice

**Figure 5:**
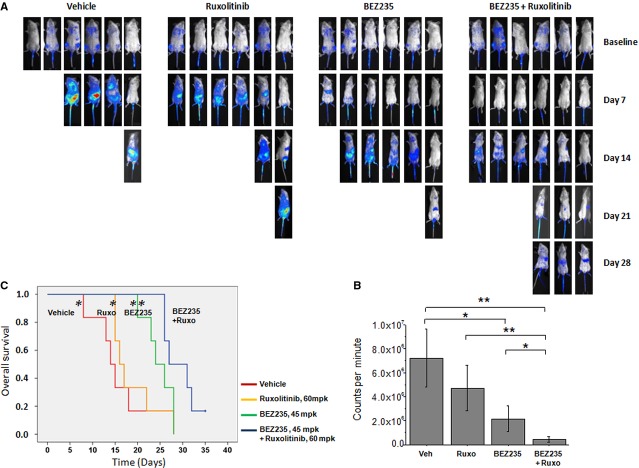
Combined treatment with BEZ235 and ruxolitinib reduces dissemination of leukaemic cells and improves survival in a *JAK2*V617F-driven mouse model. (A) SCID mice injected with 3 × 10^6^ *JAK2*V617F Ba/F3-EPOR luc^+^ cells were randomized 6 days later (baseline) into four treatment groups (vehicle, ruxolitinib [ruxo; 60 mpk], BEZ235 [45 mpk] and combination) based on baseline bioluminescence measurement. Further bioluminescence analysis was performed at weekly intervals. Images are from a representative experiment of three performed. (B) Bioluminescence activity (expressed as counts per minute, cpm; Mean ± SD of four to six mice/group in each experiment, *n* = 3) was measured on day 7 of treatment (day 13 after cells injection). (C) Kaplan–Meyer estimate of survival in mice injected with *JAK2*V617F Ba/F3-EPOR luc^+^ cells; treatment started on day 7 after injection. The survival of mice receiving combination treatment with BEZ235 and ruxolitinib was significantly improved compared with the other groups. *P < 0.05; **P < 0.01.

To corroborate the findings of synergism shown *in-vitro* by the combination of BEZ235 and ruxolitinib we used two mouse models. The first is based on the rapid, uncontrolled proliferation of Ba/F3-EPOR VF cells, stably transfected with luciferase, after systemic injection in immunodeficient mice; the progression of disease is monitored by bioluminescence at predefined time points and by measuring the survival of the animals. This represents an acute, aggressive model due to the fast growth rate and dissemination of leukemic cells with death of untreated animals occurring 10–15 days after injection. Mice were randomized to treatment groups 6 days after injection based on the bioluminescence signals, this point constitutes the baseline lecture before starting mice treatment; they received BEZ235 and ruxolitinib alone and in combination, and were followed by bioluminescence analysis at weekly intervals (Fig. 5A). In preliminary dose-finding experiments (data not shown) we determined that 50% of the animals were still alive after 15 days if receiving 120 mpk ruxolitinib and 60 mpk BEZ235 single-agent; therefore, for combination treatments described herein, we used the closest lower dose of ruxolitinib (60 mpk) and BEZ235 (45 mpk).

>Mice from the vehicle group started to die by day 7, and by day 14 less than 20% were still alive, compared with 30% of the ruxolitinib and 83% of the BEZ235 group; on the other hand, 50% of animals in theBEZ235 plus ruxolitinib combination group were still alive by day 26 when all of the mice in the other groups (vehicle, BEZ235 and ruxolitinib alone) had died. Quantitative bioluminescence measurement was performed at day 7, when at least 50% of the animals in the vehicle group were alive. At this time point, mice treated with combined BEZ235 and ruxolitinib showed significantly lower level of whole body bioluminescence readings (0.46 ± 0.23 × 10^6^ counts per minute, cpm) compared with animals receiving vehicle (7.2 ± 2.4 × 10^6^ cpm; P < 0.01), ruxolitinib (4.7 ± 1.9 × 10^6^ cpm; *P* < 0.01) and BEZ235 (2.11 ± 0.7 × 10^6^ cpm; *P* = 0.04) single agent. Of note, BEZ235 as single agent was still significantly more efficacious in comparison with vehicle (*P* < 0.05; Fig. 5B). Kaplan–Meier analysis showed that survival of mice receiving the combination of BEZ235 and ruxolitinib (median survival post-injection, 30.0 days, range 26–35) was significantly longer than mice receiving vehicle (15.0 days, range 8–18; *P* < 0.01), ruxolitinib (18.5 days, range 15–28; *P* < 0.01) and BEZ235 (24.0 days, range 20–28; *P* = 0.04) alone (Fig. 5C).

### Combination treatment of BEZ235 and ruxolitinib reduces splenomegaly and ameliorates myeloproliferation in *JAK2*V617F conditional KI mice

**Figure 6:**
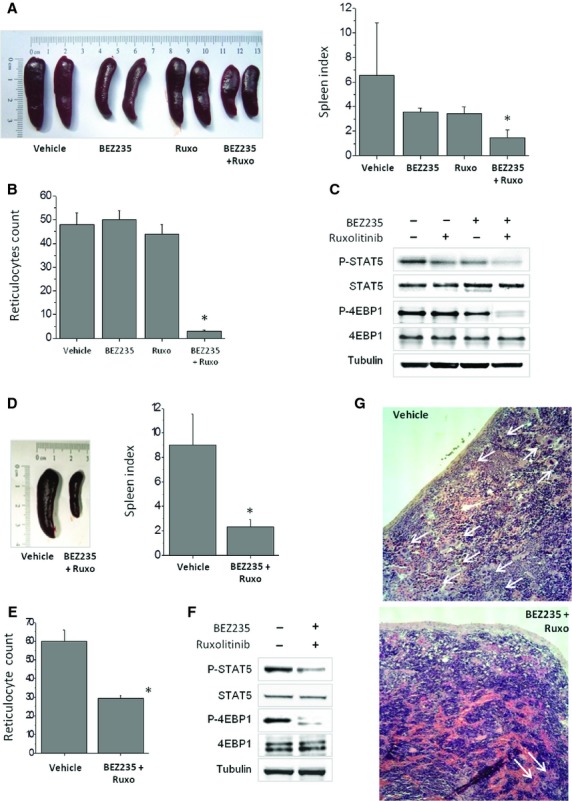
Combined treatment with BEZ235 and ruxolitinib constrains myeloproliferation in a *JAK2*V617F conditional knock-in mouse model. (A–C) *JAK2*V617F KI mice (*n* = 8/group; two experiments) were treated with BEZ235 45 mpk, ruxolitinib 60 mpk, single and in combination, or an equivalent volume of vehicle, for 7 days. In (A), on the left, representative images of the spleen at the end of treatment period, on the right the spleen index (*i.e*., spleen weight/body weight ×100) expressed as mean ± SD. In (B) the effects of treatment on reticulocyte count are shown. (C) Western blot analysis demonstrating a stronger inhibition of phosphorylated STAT5 and 4EBP1 in whole spleen extracts of mice receiving the combination of BEZ235 and ruxolitinib compared with a single agent; one representative experiment of two performed with similar results. (D–G) *JAK2*V617F KI mice were treated with the combination of BEZ235 45 mpk and Ruxolitinib 60 mpk for 16 days (*n* = 6/group). In (D), on the left, representative images of the spleen at the end of treatment period, on the right the spleen index (mean ± SD). In (E) the effects of treatment on reticulocyte count and in (F) western blot analysis of spleen extracts; one representative experiment of two performed with similar results. (G) Haematoxylin-eosin staining of the spleen of vehicle and combination treatment. Images were acquired using a LEICA DM LS2 microscope with a Leica N Plan 10×/0.25na objective and saved using Adobe Photoshop. Arrows point to single/clustered megakaryocytes, that were greatly diminished in mice treated with combination of BEZ235 and ruxolitinib. There was also a re-expression of the lymphoid component of the spleen, although the normal follicular architecture was not fully re-established.

The second *in-vivo* model was a conditional KI mouse; KI mice develop a progressive myeloproliferative disease starting from the first months after birth, characterized by marked erythrocytosis with thrombocytosis and leukocytosis, and splenomegaly, that mimics PV in early phase and evolves into myelofibrosis at later stages. In a first series of experiments, KI mice received BEZ235 and ruxolitinib alone and in combination for 7 days. We used this short lapse of time based on the observation that first effects of ruxolitinib on symptoms and splenomegaly in patients with myelofibrosis can be appreciated as early as at 2–4 weeks of treatment. We documented a prompt, dramatic reduction of spleen weight in mice receiving the two drug concurrently (Fig. 6A): the mean spleen index (*i.e*., the spleen weight normalized by the animal weight) in mice of the combination group was 1.4 compared with 6.5, 3.5 and 3.4 in mice receiving vehicle, BEZ235 and ruxolitinib alone, respectively (*P* < 0.05 for all). The mean reticulocyte count decreased from 48, 50 and 44 per HPF in the vehicle, BEZ235 and ruxolitinib group, respectively, to 3/HPF in the combination group (*P* < 0.01; Fig. 6B). Western blot analysis of whole spleen extracts documented a more pronounced decrease of pSTAT5 and p4eBP1 levels in mice receiving both drugs as compared with single drug (Fig. 6C). In a second set of experiments, animals were treated for a longer period (16 days) with the combination of BEZ235 and ruxolitinib. We confirmed the impressive reduction of splenomegaly (mean spleen index decreased from 9.0 in the vehicle to 2.3 in treated mice; *P* < 0.01, Fig. 6D), the decrease of reticulocyte count (from 60 to 29/HPF; *P* < 0.05, Fig. 6E) and the down-regulation of phosphorylated STAT5 and 4EBP1 (Fig. 6F) as seen in the short treatment set. Although not statistically significant, there was a trend towards reduced leucocyte and platelet counts in treated mice compared with vehicle group (data not shown). Finally, histology showed a marked reduction of megakaryocytes and myeloid cells infiltrating the spleen in mice treated with drug combination (Fig. 6G). Treatment was well-tolerated and the body weight loss was <10% of baseline in all treatment groups.

## Discussion

Inhibitors of the tyrosine kinase activity of JAK2 have proven efficacious in reducing splenomegaly and improving constitutional symptoms in patients with MPN, particularly MF; however, they do not selectively target the mutated clone and their potential activity is constrained by myelotoxicity, indicating that novel therapeutic strategies should be sought. In this regard, two preclinical studies with the heat shock protein-90 (HSP90) inhibitors PU-H71 40 and AUY-922 41 showed marked degradation of JAK2 in MPN cellular models, including JAK2 inhibitor-persistent cells 42, consistent with JAK2 being a client protein of the HSP90 chaperone complex; HSP90 inhibition normalized blood cell count, reduced allelic burden and improved survival in mice 40. Acetylation of HSP90 is probably involved also in the activity of histone deacetylase inhibitors, such as givinostat 43 and panobinostat 44. On the other hand, we have provided evidence that the PI3K/Akt/mTOR pathway may represent an additional suitable target for therapy; in fact, the mTOR inhibitor everolimus resulted active against *JAK2*V617F mutated cells *in vitro*, also synergizing with JAK2 inhibitors 39, and produced clinical responses in MF patients in a phase 1/2 trial 31.

The serine/threonine protein kinase Akt is downstream of PI3K 45; its main target is the serine/threonine kinase mTOR that exists in two multi-protein complexes, mTORC1 (with RAPTOR) and mTORC2 (with RICTOR). mTORC1, that is inhibited by rapalogs, phosphorylates the eukaryotic initiation factor 4E-binding protein 1 (4EBP1) and S6 kinase 1 controlling the level of cap-dependent mRNA translation. On the other hand, mTORC2 is largely rapamycin insensitive, and regulates the activity and stability of Akt by phosphorylating a conserved regulatory residue (Ser473); substrates of mTORC2 include the FOXO1 and FOXO3a transcription factors 46. Therefore, the signalling network controlled by Akt and mTOR has a central role in a variety of cellular processes that include cell growth, metabolism and proliferation; the activity of this network is elevated in most human cancers 45, including MPN, as supported by findings that: (*i*) erythroblasts of *JAK2*V617F conditional KI mice showed strong Akt activation, particularly in animals homozygous for the *JAK2* mutation 28, (*ii*) elevated levels of phosphorylated STAT5, Akt and mTOR were found in the bone marrow of MPN patients 29 and (*iii*) a strong inhibition of EEC formation and EPO-induced erythroid differentiation in PV progenitor cells was produced by a PI3K/Akt inhibitor 47. Therefore, the PI3K/Akt/mTOR pathway represents an attractive target for cancer therapy 48. Nevertheless, outside patients with renal cell carcinoma, refractory mantle cell lymphoma and ER-positive/HER2-negative breast cancer when combined with hormone therapy, the antitumour activity of the first generation of mTOR inhibitors (rapalogs) has fallen short of expectations. Reasons for this may be that these allosteric inhibitors produce incomplete inhibition of mTORC1, do not target mTORC2, and favour a rebound activation of Akt. A new generation of ATP-analog inhibitors has been developed, that include molecules targeting preferentially both mTORC1 and 2 or inhibiting also PI3K, due to similarities in the kinase domains; this double activity may be of added value for more profound inhibition of abnormal signalling in cancer cells.

In this preclinical study we have evaluated the activity of BEZ235, a dual mTOR/PI3K inhibitor that is currently under evaluation in several phase 1/2 trials, against MPN cells. Data presented indicate that mouse and human cells and cell lines expressing *JAK2*V617F are exquisitely sensitive to BEZ235 as single agent showing proliferation arrest, cell cycle blockade and also, at slightly higher concentrations, induction of apoptosis. BEZ235 also inhibited cytokine-induced clonogenic growth of MPN progenitors at concentrations significantly lower than in healthy individuals with particular efficacy against megakaryocytic progenitor probably due to the important role played in this population by PI3K pathway 30, and potently inhibited formation of EEC that are mostly derived from *JAK2*V617F mutated progenitors 10–49. The activity of BEZ235 against MPN cell lines and primary cells was synergistically enhanced by combination with the JAK1/JAK2 inhibitor ruxolitinib. Furthermore, we showed that combination of BEZ235 with ruxolitinib improved survival in an acute *JAK2*V617F-driven myeloproliferative disease in mice, and reduced splenomegaly, inhibited red cell production and improved spleen histopathology in a JAK2V617F KI mouse model.

Previous data from our laboratory showed that small molecules targeting mTORC1 (RAD001) and mTORC2 (PP242) as single agents resulted in inhibitory acivity in several *in-vitro* cellular models and, when combined with JAK2 inhibitors, produced synergistic activity 39; however, a more profound inhibition of PI3K/Akt signalling may be required for an effective anti-cancer activity. At this regard, Khan *et al*. recently reported profound inhibition of cell proliferation of MPN cell lines and reduced colony formation by primary haematopoietic progenitors of patients with myelofibrosis by using MK-2206, an allosteric inhibitor of AKT; furthermore, they were able to document alleviation of haepatosplenomegaly and reduction of megakaryocyte proliferation in a *MPL*W515L-driven MPN mouse model 50. The dual PI3K and mTOR inhibitory activity of BEZ235 may indeed be advantageous at this regard; recent *in vitro* data from Fiskus *et al*. in different MPN cell models, including cell lines that had been selected for resistance against JAK2 inhibitors, support the involvement of activated PI3K/Akt in MPN cell proliferation and survival and the effectiveness of its inhibition 51. The novel findings of a significant synergism exerted *in vivo* by combination of BEZ235 and ruxolitinib, that we report herein by using both a leukaemia model in immunodeficient mice injected with Ba/F3 cells harbouring *JAK2*V617F mutation and a *JAK2*V617F KI mouse model closely mimicking human MPN, give further strong support to the potential therapeutic relevance of dual JAK2 and PI3K/mTOR inhibition. Of relevance is also the fact that we observed strong synergistic activity in these models by using doses of the drugs that were lower than those showing activity when used as single agents. Since inhibition of normal haematopoiesis exerted by JAK2 inhibitors represents their main dose-limiting toxicity, we believe that our observations are important in the clinical setting by suggesting that enhanced activity could be obtained with lower dose of JAK2 inhibitor when used in combination with a PI3K/mTOR inhibitor, such as BEZ235, minimizing toxicity at the same time.

In summary, our findings indicate that drug-mediated inhibition of PI3K/Akt/mTOR signalling is efficacious against MPN cells and can enhance the effects of JAK2 inhibition. Therefore, concurrent targeting of the PI3K and JAK/STAT pathways may represent a new therapeutic strategy to maximize efficacy and reduce toxicity of JAK2 inhibitors that are already employed in patients with MPN.
